# Occupation-related effects on motor cortex thickness among older, cognitive healthy individuals

**DOI:** 10.1007/s00429-021-02223-w

**Published:** 2021-02-08

**Authors:** Lukas Lenhart, Melanie Nagele, Ruth Steiger, Vincent Beliveau, Elisabeth Skalla, Laura Zamarian, Elke R. Gizewski, Thomas Benke, Margarete Delazer, Christoph Scherfler

**Affiliations:** 1 Department of Radiology, Medical University Innsbruck, Anichstrasse 35, 6020 Innsbruck, Austria; 2 Neuroimaging Research Core Facility, Medical University Innsbruck, Anichstrasse 35, 6020 Innsbruck, Austria; 3 Department of Neuroradiology, Medical University Innsbruck, Anichstrasse 35, 6020 Innsbruck, Austria; 4 Department of Neurology, Medical University Innsbruck, Anichstrasse 35, 6020 Innsbruck, Austria

**Keywords:** Occupation, Cortical thickness, Neural reserve, Physical activity

## Abstract

Both, decline of sensorimotor functions and cortical thickness are known processes in healthy aging. Physical activity has been suggested to enhance the execution of daily routine activities and to extend the time of functional independence in advanced age. We hypothesized that cortical thickness of motor areas in retired individuals could be related to physical demands of the profession carried out during working life. Depending on their former occupations, 69 cognitively healthy individuals (range 70–85 years) were divided into higher and lower physically complex occupations (HPCO *n* = 27 and LPCO *n* = 42) according to the international standard classification of occupations (ISCO-08). Participants underwent a high-resolution 3T T1-weighted MRI scan. Surface-based analysis revealed higher cortical thickness in the left precentral (*P* = 0.001) and postcentral gyrus (*P* < 0.001) and right postcentral gyrus (*P* = 0.001) for the HPCO relative to the LPCO group (corrected for multiple comparisons, sex, age and leisure activities in the past 20 years). Physical leisure activities associated with exertion were positively correlated with cortical thickness in the left pre- and postcentral gyrus (*P* = 0.037) of the LPCO group. Time since retirement was negatively associated with cortical thickness in the left postcentral gyrus (*P* = 0.004) of the HPCO group. Executing a higher physically complex occupation before retirement was related to relative higher cortical thickness in the primary motor and somatosensory cortex in later life, supporting the hypothesis that physical activity contributes to neural reserve in these regions. However, these benefits appear to vanish when physical activity is reduced due to retirement.

## Introduction

The concept of brain reserve (BR) means that variations in structural characteristics of the brain allow individuals with high BR to better tolerate brain aging and pathology than people with low BR and to better preserve cognitive abilities (Medaglia et al. 2017; Stern 2017; Stern et al. 2018). This model presumes individuals with high premorbid BR to be preserved from the development of early and severe cognitive and functional impairments following brain pathology. Evidence in favor of this hypothesis has been obtained in several previous studies investigating cohorts of patients with Alzheimer’s disease (Graves et al. 1996; Stern 2002; Perneczky et al. 2010; Murray et al. 2011). The ability of the brain to learn and adapt continuously throughout life to the environment’s changing demands has been described for a broad range of cognitive activities as well as for physical- and environmental-dependent stimuli (Stern 2017). Age, genetics, and past lifetime exposures including physical activity have been shown to induce structural brain changes and to slow down age-related brain decline, a phenomenon called brain maintenance (Nyberg et al. 2012; Steffener et al. 2016).

Further to the effect of age on cognitive capabilities, sensorimotor functions have also been reported to be altered when life advances (Seidler et al. 2010). Accumulating evidence from several brain MRI studies has revealed age-related cortical decline of task-relevant motor areas as well as of brain areas that harbor sensorimotor functions such as the prefrontal cortex (for review Seidler et al. 2010; van Ruitenbeek et al. 2017). In this line, functional age-related dedifferentiation of neuronal presentation of both motor and sensorimotor systems has also been found by functional MRI (Cassady et al. 2020). These impairments include abilities of daily living such as motor control, gait and balance stability, which are all important to live an independent life. Individuals, in particular older ones, who carried out regular physical activities as a specific lifestyle component, have been shown to exhibit greater neural resources and to have an increased resilience against cortical decline and neurodegeneration (Gordon et al. 2008; Batouli and Saba 2017)*.* Furthermore, higher neural reserves in motor regions may be expected to maintain functional independence and to result in a better quality of life. In this context, the physical complexity of one’s occupation may be a determinant of cognitive and functional health and may help the brain to avert the negative effects of aging and disease (Smart et al. 2014). Occupational attainment, education and leisure activities are known to share an underlying process that differently affect cognitive domains and induce neuronal reorganization processes that remain measurable in later life (Foubert-Samier et al. 2012). In previous studies, that merely differentiated cognitively stimulating from non-stimulating activities, cognitively complex occupations were associated with higher memory and greater executive functioning (Opdebeeck et al. 2016). Thus, executing a physically complex occupation might provide an additive and independent source of neural reserve throughout life.

In consideration of the increasing proportion of older people in the society, understanding which factors might impact structural brain changes during aging becomes increasingly important. Although advanced age is the strongest known risk factor for brain atrophy, few studies focused on the influence of occupation on neural reserve. To date, it is unknown whether executing a physical demanding job modifies cortical thickness. Based on previous volumetric MRI studies that detected age-related gray matter (GM) decline in task-relevant cortical areas (van Ruitenbeek et al. 2017), we hypothesized that physical demands during an individual’s life occupation affect cortical thickness of motor areas in retirement. The objective of the present study was to assess morphological GM differences in a collective of cognitively intact (MMSE > 25), healthy participants in advanced age depending on the type of occupation performed before retirement. We further aimed to investigate the relationship between cortical thickness, physical activity at work and time since retirement in the overall cohort.

## Methods

### Study participants

Participants were independently living persons aged from 70 to 85 years, who were recruited from the general population. All individuals were right-handed, native German speakers and had normal or corrected-to-normal sight and hearing. Participants were relatives of neurological patients or recruited by word of mouth. They did not receive any monetary compensation. Inclusion criteria were a Mini-Mental State Examination (MMSE; Folstein et al. 1975) score higher than 25 and a Geriatric Depression Scale (GDS, short form; Sheikh and Yesavage 1986) score lower than 5. Participants who reported a neurological (e.g., stroke, traumatic brain injury, dementia, epilepsy), psychiatric (e.g., depression, schizophrenia, substance or alcohol abuse), major medical disease (e.g., cancer, chronic pain etc.) or contraindications against conducting a MRI (e.g., cardiac pacemaker) were excluded from participation in the study. Following inspection of MRI scans, participants with confluent white matter lesions according to Fazekas score 3 were excluded. From a total of 85 study participants, 16 were excluded due to chronic vascular leukoencephalopathy (*n* = 9), previous neurological diseases that were not reported at the MRI time-point such as severe traumatic brain injury (*n* = 1), incidental tumor finding (*n* = 1), neuropsychological test scores beyond 2 SD from the mean of standardized norms (*n* = 4) and movement artifacts (*n* = 1). The final study population consisted of 69 cognitively well-performing, healthy older participants (46 females, 23 males). The Edinburgh Handedness Inventory measurement scale was used to assess the dominance of a person's handedness in everyday activities (Oldfield 1971).

Institutional review board and written participant consent were obtained (Ethics Committee of the Medical University of Innsbruck, Austria).

### Classification of occupations

The current version of the international standard classification of occupations (ISCO-08) was used for formal categorization. Based on the amount of motor demands in previous careers, we classified individuals into either lower physically complex occupations (LPCO) or higher physically complex occupations (HPCO). The LPCO group included occupations that require interaction with people and data (including group 01: managers, 02: professionals, and 03: science and associate professionals), office, sales and care professionals (including group 04: clerical support workers, subgroup 05.2: sales workers, and 05.3: care professionals). The HPCO group comprised service workers (subgroup 05.1) and other physically challenging jobs (including group 06: skilled agricultural, forestry and fisher workers, group 07: craft and related trades workers, 08: plant and machine operators and assemblers, and 09: elementary occupations), and armed forces. We further calculated the time since retirement defined as the time period between scan date and year of retirement. Lifetime education was defined as the number of years of formal education successfully attended over a person’s lifetime and inquired during the personal interview.

### Leisure activities

Leisure activities were evaluated during a personal interview on a self-reported basis. In detail, individuals were asked for the frequency and time period of any hobbies as well as for any physically demanding or not demanding activities they had practiced over the past 20 years (Karp et al. 2006). Physical leisure activities associated with exertion included sports like walking, mountaineering, riding a bike, doing gymnastics or yoga and gardening. Bimanual activities included handcraft work such as needlework, weaving and knitting, playing an instrument with the need of two hands, doing repair works where the use of both hands is obligatory. The sum of all regularly executed physical activities associated with exertion during the last 20 years in hours per week was used for further statistical models.

### Magnetic resonance imaging data acquisition

All participants underwent high-resolution T1-weighted MRI in a 3 T scanner (MAGNETOM Skyra, Siemens Healthcare). The MRI conventional protocol included a high-resolution T1-weighted 3D MPRAGE sequence in 1 mm isotropic resolution coverage (TR 1800 ms, TE 2.22 ms, 192 contiguous coronal slices, in-plane field of view 192 × 256 mm, voxel resolution 1 × 1 × 1 mm; acquisition time 5:53 min) and an axial T2-weighted FLAIR sequence (TR = 10,000 ms, TE = 90 ms, TI = 2500 ms). All MRI data were visually inspected for artifacts arising from motion or instrument failure passed this quality control as well as the homogeneity control implemented in the CAT12 toolbox.

### Surface-based morphometry analysis

The estimation of the cortical surface was conducted using an automated processing pipeline implemented in the Computational Anatomy Toolbox (CAT, version 12.6) within SPM12 while running MATLAB 9.5 (R2018b; MathWorks, Natick, MA, USA). Briefly, high-resolution T1 images were bias-field corrected, skull-stripped, aligned to a Montreal Neurological Institute standard space (MNI-152 template) and segmented as gray matter, white matter, and cerebrospinal fluid (Ashburner and Friston 2005). The cortical thickness and the central surface were calculated in one step based on the projection-based thickness (PBT) approach, which also allowed partial volume information, sulcal blurring and sulcal asymmetries to be managed without explicit sulcus reconstruction via skeleton or thinning methods (Dahnke et al. 2013). Additionally, the surface stream included topological correction, spherical mapping, and spherical registration (Yotter et al. 2011). Finally, cortical thickness maps were re-sampled into a common coordinate system and smoothed with a Gaussian kernel of 15 mm (FWHM). The pre-processing steps were visually inspected to ensure that no misalignment of brain structures had occurred.

### Statistical analysis

Independent-sample *t *tests were performed to compare demographic variables and clinical characteristics between the LPCO and the HPCO group. A whole-brain surface-based analysis was performed to assess categorical differences between the LPCO and the HPCO group using a full factorial model with age and sex as well as physical leisure activities associated with exertion in the last 20 years and education as covariates. In addition, the interaction of education × group (LPCO, HPCO) on cortical thickness of the motor cortex was tested. Voxel-wise correlation analyses were performed by using the general linear model implemented in SPM. The relationship between MRI voxel values and physical activity as well as time since retirement were examined with *t*-contrast. Age and sex were entered as covariates. SPM analyses were performed at height-thresholds set to *P* < 0.001 for group comparisons and to *P* < 0.01 for correlation analyses and were subsequently corrected for multiple comparison of the entire brain volume using by family-wise error (FWE) correction at the cluster level (*P* < 0.05).

## Results

### Demographics and clinical characteristics

The demographics and clinical characteristics of the participants are summarized in Table 1. A total of 69 cognitively normal participants were included in this study with a mean age of 75.2 ± 3.5 years and a mean education of 11.4 ± 3.3 years. The HPCO group consisted of 27 and the LPCO group of 42 participants. Significant higher education levels were found in the LPCO compared to the HPCO group (*P* < 0.001). There were no significant differences in other demographic variables including age and sex distribution.

**Table 1:** Demographic and clinical characteristics of the entire cohort and the lower (LPCO) and higher physically complex occupation (HPCO) groups+

	Whole cohort	LPCO	HPCO
Age in years	75.2 (3.5)	75.4 (3.3)	74.9 (3.7)
Time since retirement (years)	18.4 (9.8)	17.8 (7.8)	19.2 (11.8)
Sex, % female	46 (67%)	27 (64%)	19 (70%)
MMSE	29 (0.9)	29.2 (0.8)	28.8 (1.1)
GDS	0.7 (1)	0.6 (0.9)	0.93 (1.2)
Education (years)	11.4 (3.3)	12.7 (3.2)*	9.3 (2)*
Fazekas score	1 (0.6)	1 (0.6)	1.1 (0.6)
Occupations, number			
Intellectuals		19	–
Office, sales and care professionals		23	–
Service workers		–	11
Elementary occupations		–	16
Bimanual activities per week, number			
None (0 h)	51 (74%)	36 (86%)	15 (56%)
Low (1—4 h)	6 (9%)	2 (5%)	4 (15%)
Moderate (5—9 h)	7 (10%)	3 (7%)	4 (15%)
High (> 9 h)	5 (7%)	1 (2%)	4 (15%)
Physical activities associated with exertion per week, number			
None (0 h)	5 (7%)	3 (7%)	2 (7%)
Low (1–4 h)	28 (41%)	17 (40%)	11 (41%)
Moderate (5–9 h)	30 (43%)	20 (48%)	10 (37%)
High (> 9 h)	6 (9%)	2 (5%)	4 (15%)
Physical activity associated with exertion (hours per week)	5 (5.3)	4.5 (5.4)	5.2 (4)
Total intracranial volume (TIV, mm^3^)	1451.8 (144)	1465.2 (154.4)	1431 (123.4)

Raw values are represented as mean (± 1 standard deviation). The statistical tests are corrected for multiple comparisons (Holm-Sidak) in 5% significance level

*Significant (*P* < 0.001)

### Cortical thickness of higher vs. lower physically complex occupations

**Fig. 1:**
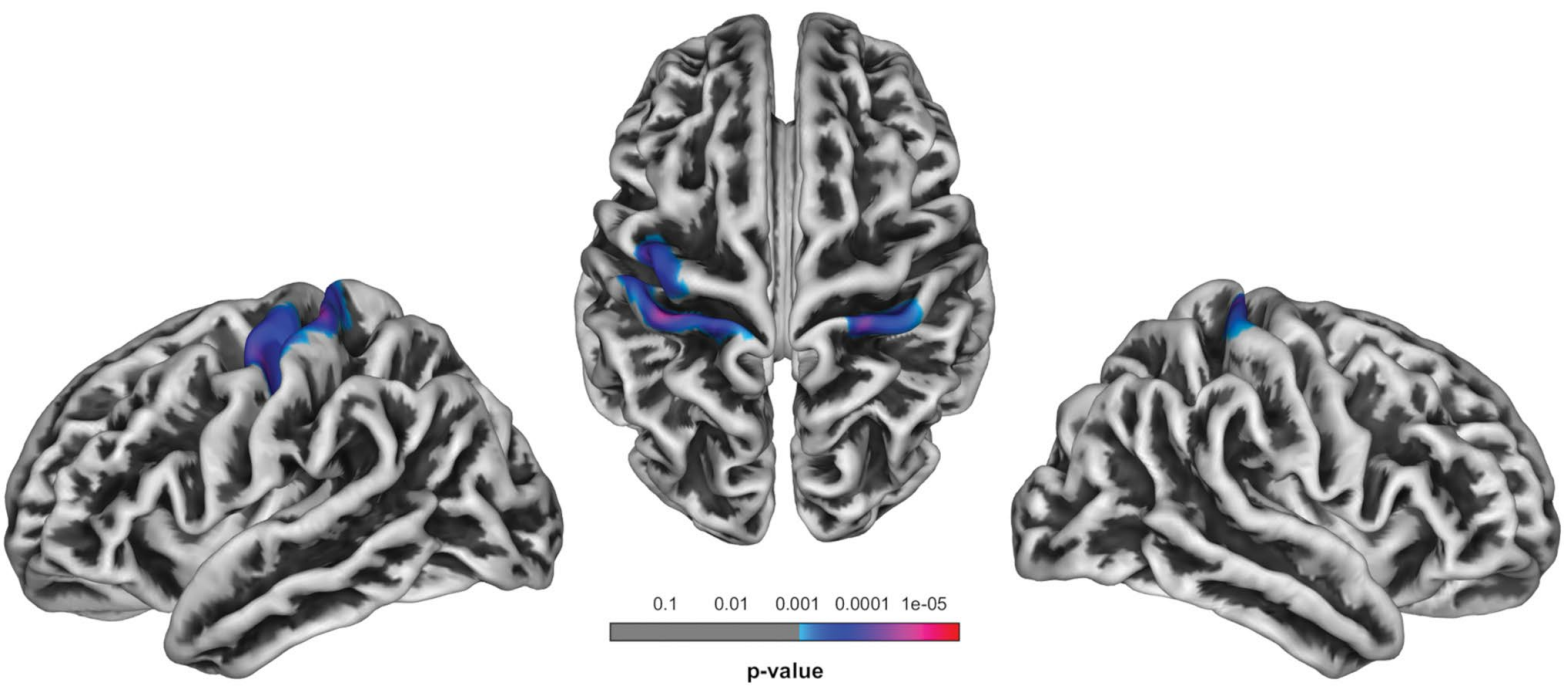
Areas of significant cortical thickness differences in the higher (27 participants) vs. the lower physically complex occupations group (42 participants); *P* < 0.001, FWE-corrected at *P* < 0.05. Cranial and lateral view

SPM localized significant higher cortical thickness in the left pre- (*P* = 0.001) and postcentral gyrus (*P* < 0.001) as well as in the right postcentral gyrus (*P* = 0.001) for the HPCO relative to the LPCO group (Table 2; Fig. 1). No significant regions of higher cortical thickness were found for the LPCO relative to the HPCO group at FWE-corrected thresholds set to *P* < 0.05. No significant interaction between the factors group and education on cortical thickness of the motor cortex was found.

**Table 2:** Overview of clusters showing significant differences in cortical thickness between higher (*n* = 27) and lower physically complex occupations (*n* = 42) in healthy individuals

Cerebral region	Cluster size	MNI coordinates			*t* value	*P* value FWE-corrected at cluster level	Height threshold
		*X*	*Y*	*Z*			
Left postcentral gyrus	482	− 34	− 30	62	4.62	< 0.001	0.001
		− 22	− 33	73			
		− 49	− 17	53			
Left precentral gyrus	323	− 40	− 13	49	3.9	0.001	
Right postcentral gyrus	300	26	− 33	70	3.93	0.001	
		37	− 29	59

### Relationships between cortical thickness, time since retirement and physical activity associated with exertion

**Fig. 2:**
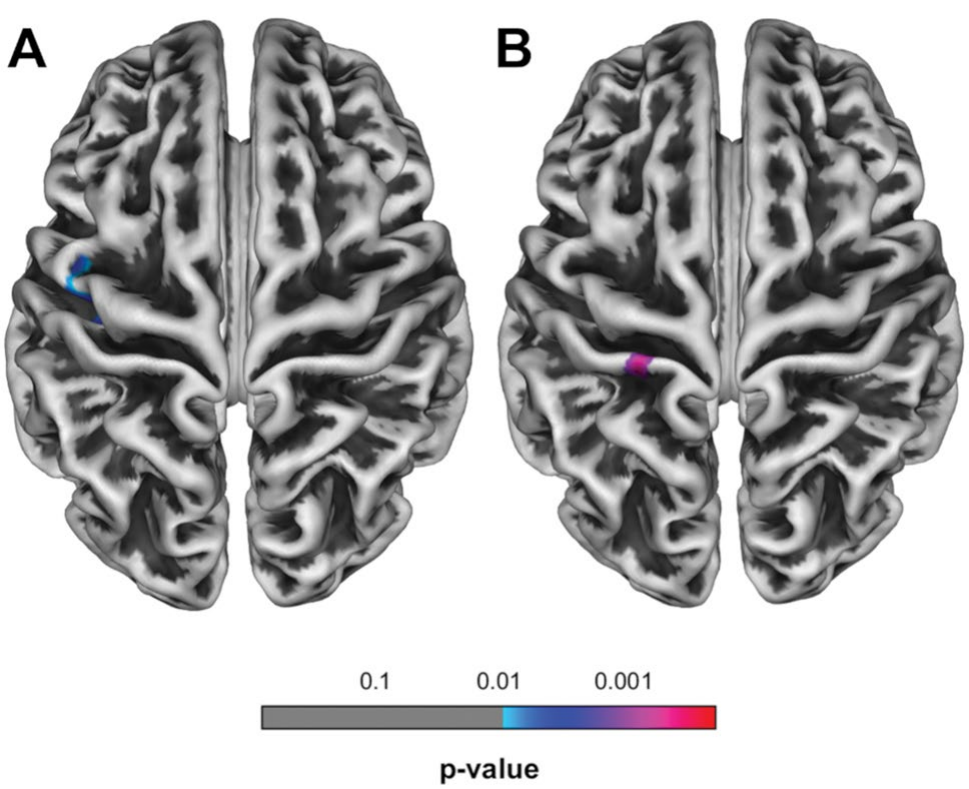
Areas of significant associations between cortical thickness in extracted clusters and **a** physical activity associated with exertion (*P* < 0.01, FWE-corrected at *P* < 0.05), and **b** time since retirement in healthy individuals (*P* < 0.01, uncorrected for multiple comparisons). Cranial view

In the LPCO group, cortical thickness measurements of a voxel cluster within the left motor cortex were positively correlated with physical activity associated with exertion (*P* = 0.037, corrected for multiple comparisons; Table 3; Fig. 2). No correlation was found between physical activity and cortical thickness in the HPCO group. Longer time since retirement was negatively correlated with cortical thickness in the left sensorimotor cortex in the HPCO group (*P* = 0.004, uncorrected for multiple comparisons; Table 3; Fig. 2).

**Table 3:** Locations of significant associations of cortical thickness in extracted clusters, physical activity associated with exertion and time since retirement in healthy individuals

Cerebral region	Cluster size	MNI coordinates			*t* value	*P* value FWE-corrected at cluster level	Height threshold
		*X*	*Y*	*Z*			
Significant correlations of cortical thickness in extracted clusters and physical activity associated with exertion in healthy individuals							
Left pre- and postcentral gyrus	213	− 48	− 20	42	3.66	0.037	0.01
		− 39	− 15	41			
		− 47	− 4	47			
Significant correlations of cortical thickness in extracted clusters and time since retirement in healthy individuals							
Left postcentral gyrus	57	− 27	− 34	68	2.87	0.004*	0.01

^*^P value at uncorrected peak level

## Discussion

In the present study comprising a cohort of 69 cognitive well-performing, healthy older individuals, we showed for the first time that executing a higher physically complex occupation before retirement is related to higher cortical thickness in specific brain areas in later life. After subclassifying the cohort and controlling for age, sex and physical leisure activities associated with exertion, individuals who had occupations with higher physical complexity revealed significantly higher cortical thickness in the primary motor and somatosensory cortex relative to people with lower physically complex occupations. Consistently with previously reported findings (Batouli and Saba 2017), we also found that the execution of physically demanding leisure activities is associated with higher cortical thickness in primary motor and somatosensory regions in the LPCO group, supporting the hypothesis that exercise supports maintenance of neural resources in these regions of retired individuals with lower physically complex occupations during working life.

Regions that are involved in movement and motor control, particularly the precentral cortex and the basal ganglia, were shown to be particularly vulnerable to aging processes (Seidler et al. 2010). Several studies have pointed out that cortical thickness in primary motor regions is correlated with the level of cardiorespiratory fitness (Weinstein et al. 2012; Williams et al. 2017), even after 1 week of motor adaption training (Landi et al. 2011). Previous analyses have also shown that neuroplasticity in pre- and postcentral cortices is modifiable through gymnastics (Huang et al. 2018) and balance training (Taubert et al. 2016; Rogge et al. 2018). Additional evidence comes from studies comparing groups executing bimanually activities to control cohorts. For instance, increased GM volume was found in motor as well as auditory and visuospatial brain regions in professional musicians (keyboard players) compared to amateur musicians and non-musicians (Gaser and Schlaug 2003). In a further study comprising a cohort of 44 naïve juggling participants, better performance was correlated with GM increases in the parietal and motor cortices suggesting that practice time and performance can modulate the degree of structural brain change evoked by long-term training regimes (Sampaio-Baptista et al. 2014). We assume that executing a physically demanding job initiates a similar training effect and results in higher cortical thickness in motor regions. Volume loss in task-relevant cortical areas was shown to be negatively associated with complex bimanual coordination performance (van Ruitenbeek et al. 2017). In this line, another study of elderly adults revealed increased activation of the somatosensory cortex which correlated with motor performance (Goble et al. 2010). Especially in individuals with advanced age, who were frequently affected by motor performance deficits, increased cortical thickness in the somatosensory cortex was suggested to represent greater proprioceptive information processing of the hands to maintain the desired coordination function (Goble et al. 2010).

It is important to mention that the rate of age-related decline varies between different brain areas. In this context, the precentral cortex was reported to degenerate more rapidly compared to other regions (Pfefferbaum et al. 2013). In the present study, time since retirement was significantly correlated with cortical decline in the HPCO group, but not in the LPCO group. This fits well with previous analyses that measured cortical decline in non-demented, healthy individuals and detected age-related atrophy patterns prominently in primary motor cortices using larger cohorts (Salat et al. 2004; Sowell et al. 2003) or a within-subject design (Pfefferbaum et al. 2013; Raz et al. 2005). We assume that after retirement and the consequently absence of regular physical activity, higher neural reserve in the HPCO group decreases quicker to a similar level as in the LPCO group. In this line, it was observed that individuals with physically demanding occupations prefer physically more passive leisure activities and vice versa (Lakka et al. 1996; Finger et al. 1998). This inversion of preferences might mitigate the differences in cortical thickness following retirement. Frequent physical exercises may lower the steepness of this slope and help to maintain acquired structural reserves in motor regions. Concordantly, the results of a recent review demonstrated that physical activity is associated with a large network of brain areas comprising 82% of the total GM volume (Batouli and Saba 2017). Furthermore, physical exercise was shown to upregulate the expression of cytokine interleukin (IL)-10 and attenuate levels of IL-ß and tumor necrosis factor alpha (TNFα) secreted from reactive astrocytes and microglia, which was hypothesized to result in an anti-inflammatory environment within the brain (Kelly 2018). The modulation of neuroinflammatory processes through physical exercise seems to induce a reversal of deficits in the adult neurogenesis and synaptic plasticity, and to slow down cellular and cognitive impairments (Shepherd et al. 2018). Physical activity appeared to be a propitious trigger to maintain overall GM volume in late adulthood, especially in frontal and temporal cortices (Bugg and Head 2011), the hippocampus (Head et al. 2012) and the prefrontal cortex (Weinstein et al. 2012; for review Erickson et al. 2014).

This study has some limitations. The categorization of occupations was based on the ISCO-08 classification and might not accurately reflect the unique physical demands of everyone’s occupation. Another limitation is that our study design did not include physical examinations, such as a bimanual tracking task, and therefore does not allow the assessment of functional correlates. Further longitudinal studies in older adults with detailed physical testing are required to confirm our results and to determine potential protective and therapeutic effects of exercise on cortical thickness.

## Conclusions

The present study provides for the first time evidence that people over the age of 70 differ in structural reserve depending on the physical complexity of their occupation during working life. Since individual’s occupation represents a large part of adult’s life, regular physical activity is strongly suggested to maintain and strengthen cortical thickness in primary motor and somatosensory regions, which in turn may preserve motor abilities in older age. However, the protective effects appear to vanish if continuous physical activities were reduced as a consequence of retirement.

## Data Availability

Data will be shared with qualified parties.
